# KAP1 Deacetylation by SIRT1 Promotes Non-Homologous End-Joining Repair

**DOI:** 10.1371/journal.pone.0123935

**Published:** 2015-04-23

**Authors:** Yi-Hui Lin, Jian Yuan, Huadong Pei, Tongzheng Liu, David K. Ann, Zhenkun Lou

**Affiliations:** 1 Department of Biochemistry and Molecular Biology, Mayo Graduate School, Rochester, Minnesota, United States of America; 2 Research Center for Translational Medicine, Tongji University School of Medicine, Shanghai, China; 3 Key Laboratory of Arrhythmias of the Ministry of Education of China East Hospital, Tongji University School of Medicine, Shanghai, China; 4 State Key Laboratory of Proteomics, Beijing Proteome Research Center, Beijing Institute of Radiation Medicine, Beijing, China; 5 Division of Oncology Research, Department of Oncology, Mayo Clinic, Rochester, Minnesota, United States of America; 6 Department of Molecular Pharmacology and Irell and Manella Graduate School of Biological Sciences, Beckman Research Institute, City of Hope, Duarte, California, United States of America; St. Georges University of London, UNITED KINGDOM

## Abstract

Homologous recombination and non-homologous end joining are two major DNA double-strand-break repair pathways. While HR-mediated repair requires a homologous sequence as the guiding template to restore the damage site precisely, NHEJ-mediated repair ligates the DNA lesion directly and increases the risk of losing nucleotides. Therefore, how a cell regulates the balance between HR and NHEJ has become an important issue for maintaining genomic integrity over time. Here we report that SIRT1-dependent KAP1 deacetylation positively regulates NHEJ. We show that up-regulation of KAP1 attenuates HR efficiency while promoting NHEJ repair. Moreover, SIRT1-mediated KAP1 deacetylation further enhances the effect of NHEJ by stabilizing its interaction with 53BP1, which leads to increased 53BP1 focus formation in response to DNA damage. Taken together, our study suggests a SIRT1-KAP1 regulatory mechanism for HR-NHEJ repair pathway choice.

## Introduction

Homologous recombination (HR) and non-homologous end joining (NHEJ) are two distinct pathways for repairing DNA double-strand-break (DSB), which is the most lethal cytotoxic lesion in response to genotoxic stress. HR-mediated repair resects DNA sequence near damage sites, and then follows the guide of homologous sequence on the sister chromatid to restore the DNA lesions precisely [1–3]. In contrast to the template-requiring mechanism, NHEJ mediates an error-prone repair process that directly ligates DSBs [4,5]. Cells under different cellular conditions have developed selective preferences for HR or NHEJ pathways in response to DSBs. For instance, HR functions in S and G2 phases due to the availability of sister chromatids. There is also evidence suggesting that HR plays a critical role in one-sided DSBs caused by collapsed replication fork [6,7]. In contrast to the cell phase specificity of HR, NHEJ pathway is active throughout the cell cycle, with a major contribution during the G1 phase [8,9]. In order to optimize the balance between HR and NHEJ, additional mechanisms have been adopted to regulate DSB repair pathway choice without direct involvement in the catalytic steps of DNA repair. BRCA1 is an E3-ubiquitin ligase that interacts with DNA repair proteins, such as CtBP-interacting protein (CtIP) and MRE11-RAD50-NBS1 (MRN) complex, and facilitates the 5’ end resection during HR [10–16]. Indeed, resection at DSBs and RAD51 focus formation were impaired in the absence of BRCA1 [17–19], which lead to defect in HR repair [12,20]. These studies demonstrate the critical role of BRCA1 in promoting HR. By contrast, p53-binding protein 1 (53BP1) has been reported as an important NHEJ promoting factor. Upon DNA damage, 53BP1 localizes to DSB sites through recruitment of mono- and dimethyl- H4K20 and the RNF168-ubiquitylated H2A-K15 [21–23]. Additionally, 53BP1 is phosphorylated on the N-terminal region by ATM, which assists the binding to other effectors including Rap1-interacting factor 1 homolog (RIF1) and Pax transactivation-domain interacting protein (PTIP) [14,15,24–35]. As a result, binding of 53BP1 protein complex to the DNA damage sites blocks 5’ resection, thereby promoting NHEJ-mediated repair. [36]. Although BRCA1 and 53BP1 function in distinct pathways, genetic interaction between these two factors has been demonstrated by several studies [16,37,38], suggesting that 53BP1 and BRCA1 may counteract with each other to regulate the DNA repair pathway choice.

Sirtuin 1 (SIRT1), a nicotinamide adenosine dinucleotide (NAD^+^)-dependent deacetylase, is widely recognized as a critical regulator in metabolic disease, aging, and cancer development [39–41]. Reports have shown that SIRT1 activity increases upon nutrient deficiency and calorie restriction [42–44]. SIRT1 also participates in epigenetic regulation by deacetylating histones and chromatin modifiers [45–47]. SIRT1 deacetylates p53 and Forkhead box O (FOXO) family of proteins, preventing cells from undergoing cell cycle arrest and apoptosis [48–51]. Furthermore, SIRT1 promotes DNA repair capacity by deacetylating repair proteins such as Ku70, Nijmegen breakage syndrome protein (NBS1), Werner syndrome protein (WRN), and Xeroderma pigmentosum complementation group A (XPA) [52–55]. Additionally, there are studies demonstrate that SIRT1 also plays a distinct role in DSB repair pathway selection [56–59]; however, the detailed mechanism remains poorly understood.

Krüppel associated box (KRAB)-associated protein 1 (KAP1) is a universally encoded transcriptional co-repressor. The tripartite RBCC (RING, b-box, coiled-coil) domain on the N-terminus of KAP1 can selectively bind to the KRAB domain of zinc finger protein-based transcription factors. The PxVxL motif and the PHD-Bromo domain on the C-terminus are responsible for recruiting chromatin modifiers, such as heterochromatin protein 1 (HP-1), Histone-lysine N-methyltransferase SETDB1, nucleosome remodeling and deacetylation (NuRD) complex, and histone deacetylase (HDAC) family of proteins, to induce downstream transcriptional repression [60–63]. Based on its capability to regulate various chromatin-remodeling complexes, KAP1 is proposed to be a master regulator for genomic stability, heterochromatin formation, and target gene silencing. In addition, KAP1 also plays an essential role in the DNA damage response (DDR). Upon DNA damage, KAP1 is rapidly phosphorylated by Ataxia telangiectasia mutated (ATM) on Ser824, which leads to chromatin relaxation and promotes efficient DNA repair [64]. Furthermore, phosphorylation of Ser824 (pSer824) abolishes the SUMOylation in bromo domain, and thus relieves the repression on genes involved in cell cycle arrest and pre-apoptotic signaling, such as *p21*, *Bax*, *PUMA*, and *Noxa* [65–67]. These studies demonstrate that post-translational modification plays an essential role in assisting KAP1 to execute pleiotropic effects in the DDR.

Here we report that KAP1 is a substrate of SIRT1 and plays a role in selection of DNA damage repair pathway. We show that while KAP1 acts as a negative regulator of HR, it enhances the NHEJ-mediated repair. Mechanistically, we demonstrate that the deacetylation of KAP1, which is regulated by SIRT1, stabilizes the protein-protein interaction between KAP1 and 53BP1, thereby enhancing the NHEJ-mediated repair efficiency in a pSer824 independent manner. In summary, our study suggests a SIRT1-KAP1 regulatory mechanism for DSB repair pathway choice.

## Materials and Methods

### Cell culture and plasmids

HEK293T (ATCC, Cat No. CRL-11268) cells were cultured in Dulbecco's Modified Eagle Medium (Invitrogen, Cat No. 11965–092) supplemented with 10% fetal bovine serum (FBS) (Atlantic Biologicals, Cat No. S11150). U2OS (ATCC, Cat No. HTB-96) cells were cultured in McCoy’s 5A Medium (HyClone, Cat No. SH3020001) with 10% FBS supplemented. KAP1 mutant was cloned into pIRES-EGFP and pCMV-HA vectors. 4KR acetylation null mutant was generated with site-directed mutagenesis (Stratagene) and verified by sequencing. Primers used for generating 4KR mutant are described in Supporting Information (S1 Table). WT and mutant SIRT1 were previously described [68]. GAL4-tagged KAP1 truncated mutants and pCMV-tag2B KAP1 phospho mutants (S824A and S824D) [67] were kindly provided by Dr. David Ann (Beckman Research Institute).

### Chemicals and Antibodies

SIRT1 selective inhibitor (Ex527) was purchased from Sigma (Cat No. E7034) and diluted in dimethyl sulfoxide (DMSO) to 20 mg/mL as stock solution. Commercial antibodies were used to detect anti-acetylated lysine (Rockland, rabbit polyclonal, Cat No. 600-401-939), anti-GAL4 DNA binding domain (Millipore, rabbit polyclonal, Cat No. 06–262), anti-BRCA1 (Santa Cruz, mouse monoclonal, Cat No. sc-6954), anti-FLAG (Sigma, mouse monoclonal, Cat No. F3165), anti-HA (Sigma, rabbit polyclonal, Cat No. H6908/ mouse monoclonal, Cat No. H3663), and anti-β-actin (Sigma, mouse monoclonal, Cat No. A2228). Homemade antibodies against SIRT1, KAP1, phospho-KAP1 (Ser824), and 53BP1 were raised by immunizing rabbits with SIRT1-peptide (724–747 a.a), GST-KAP1 (697–835 a.a), phospho-KAP1 (824)-peptide (GAGLS(pS)QELSG), and GST-53BP1 (338–671 a.a).

### RNA Interference

SIRT1 and KAP1 shRNAs targeting 3’UTR region were purchased from Sigma. The sequence for SIRT1 3’UTR shRNA was 5’- GCAAAGCCTTTCTGAATCTAT- 3’. The sequence for KAP1 3’UTR shRNA was 5’- CCTGGCTCTGTTCTCTGTCCT- 3’.

### *In Vitro* SIRT1 Deacetylase Assay

*In vitro* deacetylation assay was performed as described previously [68]. Briefly, FLAG-tagged KAP1 and HA-tagged p300 were transiently expressed in HEK293T cells. FLAG-tagged SIRT1 was transfected separately. Recombinant KAP1 and SIRT1 were immunoprecipitated by anti-FLAG M2 Affinity Gel (Sigma, Cat No. A2220) and eluted with 100μg/ml 3X FLAG peptide (Sigma, Cat No. F4799) in Tris-HCl (pH 8.0). Acetylated KAP1 was incubated with 50μM NAD^+^ and recombinant SIRT1 in deacetylase buffer (50mM Tris-HCl, pH9.0, 5% glycerol, 50mM NaCl, 4mM MgCl_2_, 0.5mM DTT, 0.1mM PMSF, 0.02% NP-40) in the presence or absence of Ex527 (40 μM) for 90min at 30°C. Total acetylation level of KAP1 was examined by immunoblotting.

### Luciferase Assay

Cells (25,000/well) were plated in 24-well plates the night before transfection. Each well was transfected with a mixture containing 100ng E2F1-responsive (Cyclin D3) luciferase reporter vector, 100ng E2F1 expression vector, 350ng pCMV-HA KAP1 or 4KR expression vectors, 20ng pCMV-HA vector, and 50ng pRL-TK vector. Transfection was performed using Lipofectamine 2000 reagent (Life Technologies), and cells were analyzed for luciferase and renilla activity after 36 hours. The ratio of luciferase/renilla activity was used as the indicator for transcriptional activation. The E2F1-responsive luciferase reporter vector and E2F1 expression vector were kindly provided by Dr. Jiandong Chen (Moffitt Cancer Center).

### HR Assay

HR assay was performed as described previously [69]. Briefly, HEK293T cells were plated in 6-well plates and transfected with a mixture containing *DR-GFP* reporter, I-*Sce*I expression vector (pCBA-I-*Sce*I), and expression vectors for reconstitution. Transfection was performed using Lipofectamine 2000 reagent. Cells were harvested 48 hours after transfection and examined by flow cytometric analysis. To compare the recombination efficiency, pIRES-EGFP was transfected similarly and used to normalize for transfection efficiency.

### NHEJ Assay

The *in vivo* end-joining reporter pEGFP-Pem1-Ad2 has been previously described [70]. Prior to transfection, the reporter vector was digested with HindIII at 37°C overnight. Cells were plated in 6-well plates and transfected with linearized pEGFP-Pem1-Ad2 reporter and expression vectors for reconstitution. 48 hours after transfection with Lipofectamine 2000 reagent, cells were harvested for flow cytometric analysis. pIRES-EGFP was transfected similarly and used to normalize for transfection efficiency.

### Immunofluorescence Staining

U2OS cells were grown on coverslips and fixed with 3% paraformaldehyde for 10 minutes, permeabilized in 0.5% Triton-X for 5 minutes at room temperature. Blocking was done in 5% goat serum at room temperature for 1 hour. Cells were incubated with primary antibody at 37°C for 20 min, followed by incubation of Alexa Fluor 488 or rhodamine-conjugated secondary antibodies at 37°C for 20 min. Nuclei were counterstained with DAPI. Slides were washed with PBS between incubations.

### Statistics

Experiments with three replicates were performed. Statistical analysis was carried out with Student's t-test (two-tailed). Values with P < 0.05 were considered significant.

## Results

### KAP1 is a novel binding partner of SIRT1 complex

**Fig 1:**
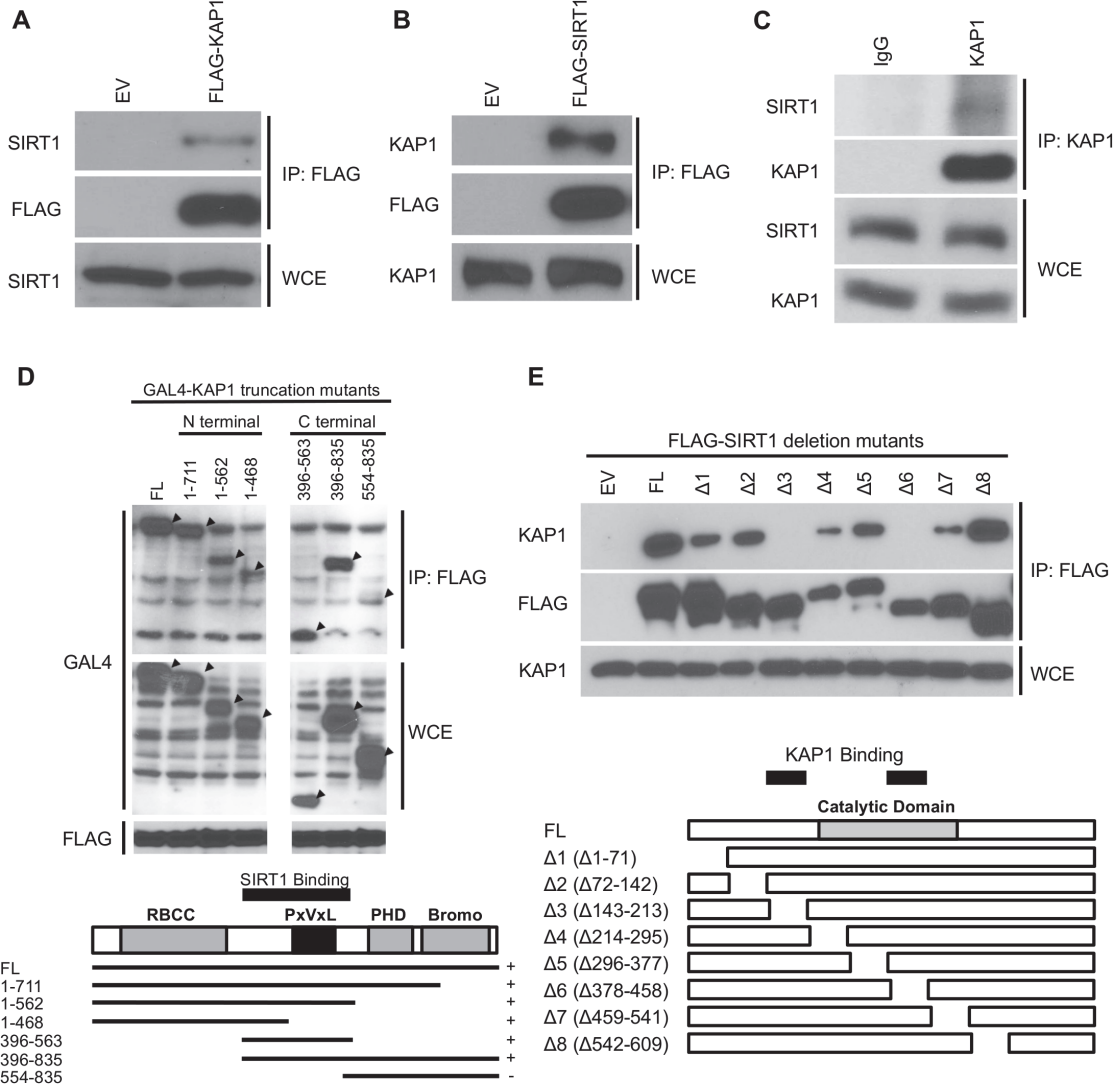
KAP1 is a binding partner of SIRT1 complex. (A and B) HEK293T cells transfected with empty vector, FLAG-tagged KAP1, or FLAG-tagged SIRT1 were lysed and immunoprecipitated with anti-FLAG antibody. Whole cell extracts (WCE) and immunoprecipitates were blotted with the indicated antibodies. (C) HEK293T cell lysates were subjected to immunoprecipitation with control IgG or anti-KAP1 antibody. The SIRT1-KAP1 interaction was examined by western blot using anti-SIRT1 antibody. (D) FLAG-tagged SIRT1 was co-transfected with GAL4-tagged full-length (FL) KAP1 or the truncation mutants of KAP1 into HEK293T cells, followed by immunoprecipitation with anti-FLAG antibody. The interaction between KAP1 truncation mutants and full-length SIRT1 was determined by western blot analysis. (E) FLAG-tagged full-length (FL) SIRT1 or the SIRT1 deletion mutants were transfected into HEK293T cells and immunoprecipitated with anti-FLAG antibody. Immunoprecipitates were analyzed by anti-KAP1 antibody.

SIRT1 was previously described to play a direct or indirect role in DNA repair process [48–54,71]. In order to study the unknown function of SIRT1 in DNA repair pathway, we were prompted to explore the novel SIRT1 interacting proteins. Immunoprecipitation assay was performed in HEK293T cells with FLAG-tagged SIRT1 and followed by mass spectrometric analysis to identify potential SIRT1 binding partners [72]. In addition to other known SIRT1 interacting factors, we found KAP1 as the major binding partner of SIRT1 associated complex. Interestingly, similar result was previously described in other published work [73]. We then confirmed this SIRT1-KAP1 interaction by performing co-immunoprecipitation experiments. As shown in Fig 1A and 1B, we observed the *in vivo* interaction of SIRT1-KAP1 using either FLAG-tagged KAP1 or SIRT1. The same result could be acquired by immunoprecipitating endogenous KAP1 (Fig 1C).

In order to map the SIRT1-interacting region of KAP1, we used the GAL4-tagged KAP1 truncation mutants to pull down full-length SIRT1 protein. Our results showed that loss of a.a 396−554 of KAP1 abolished its interaction with SIRT1, suggesting this PxVxL containing region of KAP1 is essential for SIRT1 association (Fig 1D). On the other hand, a set of FLAG-tagged SIRT1 deletion mutants was used to characterize the KAP1-interacting region of SIRT1 [68]. In this experiment, SIRT1 deletion mutants were transfected into HEK293T cells and co-immunoprecipitation experiments for KAP1 were performed. We found that SIRT1 mutants with deletion in 143–213 a.a (Δ3) or 378–458 a.a (Δ6) could completely abolish the binding, suggesting that these regions on SIRT1 are required for KAP1 interaction (Fig 1E). Collectively, these results suggest that KAP1 is a novel binding partner of SIRT1 complex. In addition, two regions of SIRT1 (143–213 a.a and 378–458 a.a) and 396–554 a.a of KAP1 are necessary for the protein-protein interaction.

### SIRT1 deacetylates KAP1 *in vitro* and *in vivo*

**Fig 2:**
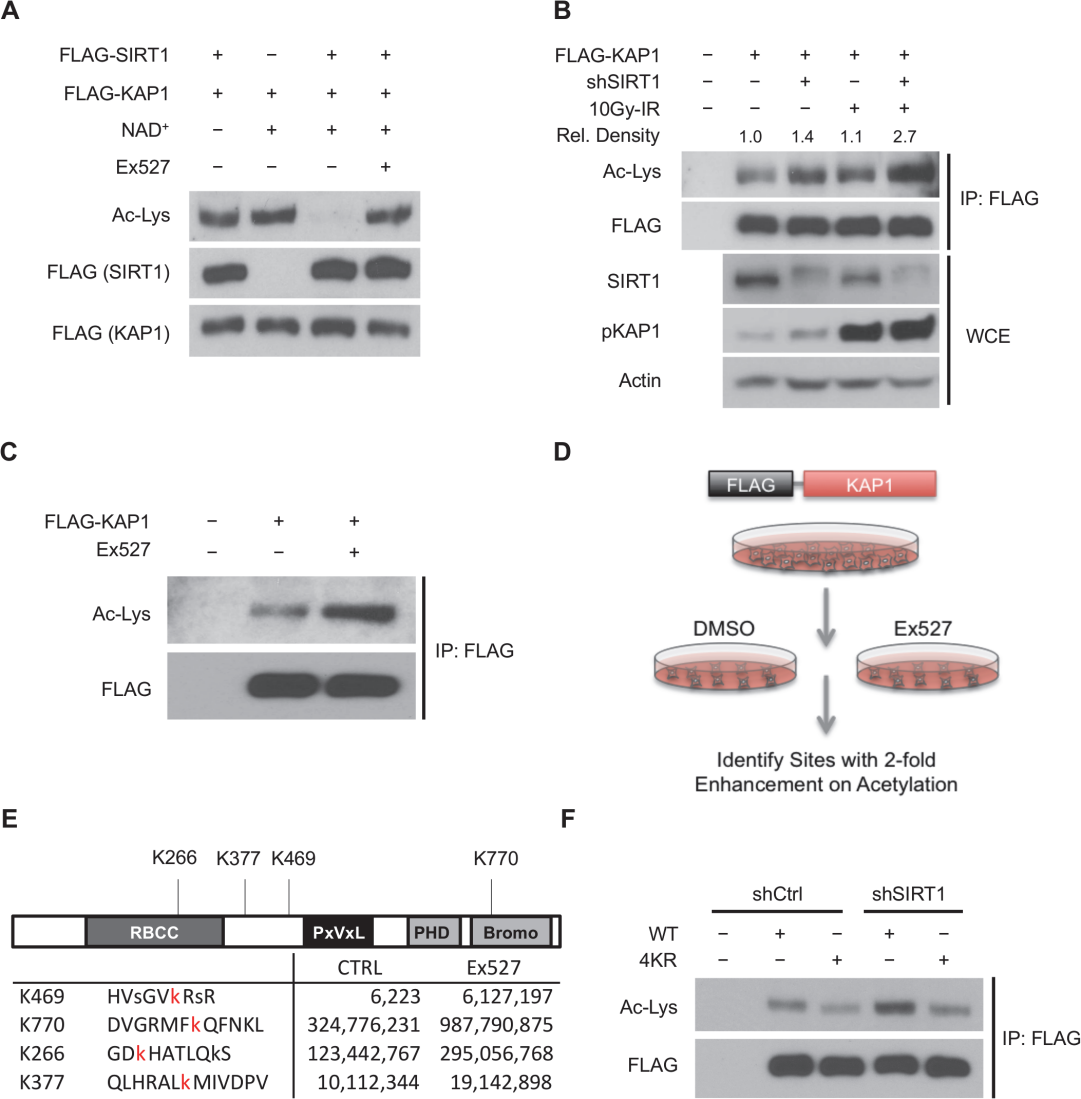
SIRT1 deacetylates KAP1 *in vitro* and *in vivo*. (A) SIRT1 deacetylates KAP1 *in vitro*. Exogenous FLAG-tagged KAP1 and SIRT1 were purified by anti-FLAG immunoprecipitation. Combination of purified proteins was incubated in deacetylation buffer supplemented with or without NAD^**+**^ cofactor. Ex527 (40μM) was added to block the deacetylase activity of SIRT1. (B) SIRT1 deacetylates KAP1 *in vivo*. FLAG-tagged KAP1 was transfected into control or SIRT1 depleted HEK293T cells. Cells were treated with or without IR 1 hour before harvest. Cell lysates were subjected to immunoprecipitation using anti-FLAG antibody, followed by western blot analysis to assess the total KAP1 acetylation level. Relative density of the overall acetyl lysine was quantified using ImageJ software. Acetylation level was normalized to corresponding FLAG band. (C) HEK293T cells transfected with FLAG-tagged KAP1 were treated with DMSO or Ex527 (20μM) for 6 hours before harvest. Cell lysates were then immunoprecipitated with FLAG-conjugated agarose beads, and the immunoprecipitates were blotted with anti-acetyl lysine antibody to determine the total acetylation level. (D and E) HEK293T cells were transfected with FLAG-tagged KAP1 and treated with DMSO or Ex527 (20μM) for 6 hours before harvest. Recombinant KAP1 was purified and sent for mass spectrometric analysis. Acetyl residues with >2-fold enhancement after inhibitor treatment were considered to be SIRT1 targeted sites. (F) Site-directed mutagenesis was applied to generate 4KR mutant. FLAG-tagged WT-KAP1 or 4KR mutant were transfected into control or SIRT1 depleted HEK293T cells. Total acetylation levels of recombinant WT-KAP1 and 4KR mutant were assessed by immunoblotting.

The interaction between SIRT1 and KAP1 raised the possibility that KAP1 is a catalytic substrate of SIRT1. To examine the potential role of SIRT1 in regulating KAP1 acetylation, we investigated whether SIRT1 could deacetylate KAP1. As shown in Fig 2A, SIRT1 deacetylated KAP1 in the presence of NAD^+^ cofactor *in vitro*. The deacetylation was reversed by adding Ex527, a SIRT1 selective inhibitor, suggesting that SIRT1 activity is responsible for KAP1 deacetylation *in vitro* [74]. To explore the SIRT1-dependent deacetylation *in vivo*, we analyzed total KAP1 acetylation in control or SIRT1 depleted cells, and we found that depletion of SIRT1 moderately enhanced KAP1 acetylation (Fig 2B). Additionally, to test whether KAP1 acetylation is responsive to DNA damage, we treated cells with ionizing radiation (IR) and examined KAP1 acetylation following DSB. To our surprise, SIRT1 depleted cells demonstrated strong enhancement on KAP1 acetylation after IR treatment (Fig 2B), suggesting that SIRT1 plays a role in regulating KAP1 acetylation following DNA damage. Although the total KAP1 acetylation level in wild type cells changed subtly after IR, it might due to the interference from other acetyl residues in the background [75]. In order to verify whether this regulation is dependent on the enzyme activity of SIRT1, we treated cells with Ex527 and examined the total acetylation level of KAP1. We found that KAP1 acetylation was increased dramatically after treating with SIRT1 inhibitor (Fig 2C). In summary, these results suggest that SIRT1 deacetylates KAP1 in response to DNA damage.

Next, to identify the potential SIRT1 regulated KAP1 acetylation site(s), cells were treated with Ex527 and followed by acetyl-specific mass spectrometric analysis (Fig 2D). We found that treatment with Ex527 significantly enhanced acetylation on four lysine residues (Lys266, Lys377, Lys469, and Lys770) (Fig 2E). These sites were also identified in a previously established acetylome, although the regulation of those sites was unclear [75]. By alignment analysis, we found that these four lysine sites are conserved in mammals; moreover, Lys266 and Lys377 are even conserved in more distant species (S2 Table). Consistent to the mass spectrometric results, we found that KAP1 acetylation was lower when these four lysine residues were mutated to arginine (4KR) (Fig 2F). It is noteworthy that the 4KR mutant still preserved detectable acetylation background, suggesting some acetyl sites are regulated in a SIRT1-independent manner. Importantly, while depletion of SIRT1 significantly increased the acetylation on wild-type KAP1 (WT-KAP1), the change on 4KR mutant was minimal. Taken together, these results indicate that Lys266, Lys377, Lys469, and Lys770 are the major SIRT1-regulated acetylation sites on KAP1.

### Acetylation does not crosstalk with IR-induced pSer824 and does not affect the co-repressor activity of KAP1

**Fig 3:**
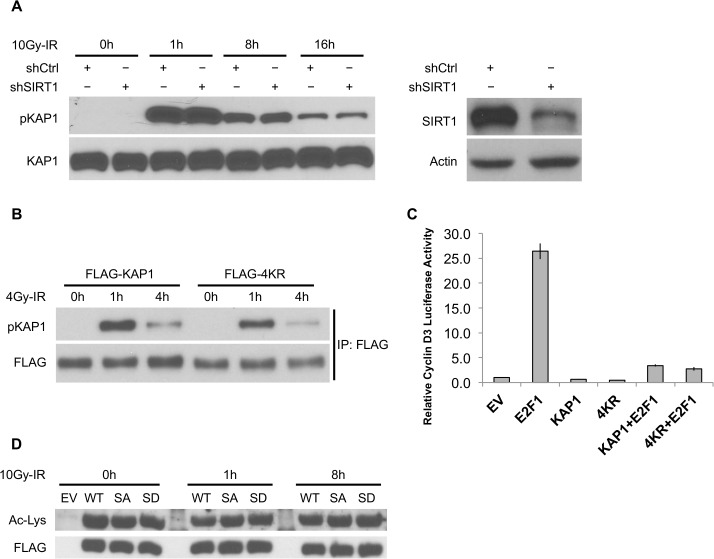
Acetylation does not crosstalk with IR-induced pSer824 and does not affect the co-repressor activity of KAP1. (A) Control and SIRT1 depleted HEK293T cells were harvested at indicated time points following 10Gy-IR. IR-induced KAP1 pSer824 was examined by phosphorylation specific antibody. (B) Cells transfected with FLAG-tagged WT-KAP1 or 4KR mutant were harvested at indicated time points following 4Gy-IR. Exogenous KAP1 was immunoprecipitated and pSer824 was examined by phosphorylation specific antibody. (C) Cyclin D3 luciferase construct was co-transfected with combinations of HA-tagged E2F1, FLAG-tagged KAP1, and 4KR mutant. Luciferase activity was measured 48 hours post transfection. (D) Cells transfected with FLAG-tagged WT-KAP1 or KAP1 phospho mutants (S824A and S824D) were harvested at indicated time points following 10Gy-IR. Recombinant KAP1 was purified by immunoprecipitation and the total acetylation level was assessed by immunoblotting.

In response to DNA damage, SIRT1 promotes cell survival by deacetylating target transcription factors and attenuating their activities to trans-activate cell-cycle arrest and apoptosis [48,49]. As a prominent substrate of ATM, KAP1 is phosphorylated on Ser824 in response to genomic stresses, presumably to increase the DNA repair capacity and de-repress downstream genes that regulate proliferation and survival [76–78]. Given the known role of KAP1 in transcription and DNA repair, we investigated whether SIRT1 regulates the function of KAP1. Based on previous findings that KAP1 responds to DNA damage through pSer824, we first examined the induction of pSer824 in SIRT1 depleted cells. Both in wild type and SIRT1-depleted cells, we found significant and comparable enhancement of KAP1 pSer824 in response to IR-induced DNA damage (Fig 3A). Similarly, induction of pSer824 was comparable on WT-KAP1 or 4KR mutant after IR stimulation (Fig 3B). These results demonstrate that KAP1 acetylation does not affect KAP1 pSer824 following DNA damage. Furthermore, we assessed the role of acetylation in regulation of KAP1 co-repressor activity. A previously described E2F1-responsive promoter was used to determine the activity of KAP1 in transcription [79]. E2F1 expression vector and E2F1-responsive promoter (Cyclin D3) were co-transfected into HEK293T cells. We found that overexpressing either WT-KAP1 or 4KR mutant could suppress E2F1 transcriptional activation to the same extent (Fig 3C), suggesting that KAP1 deacetylation does not affect its transcriptional co-repressor function. On the other hand, when we examined the acetylation level of KAP1 phospho mutants (S824A and S824D), we didn’t observe apparent change in acetylation compare to WT-KAP1 (Fig 3D), suggesting no crosstalk takes place between KAP1 phosphorylation and acetylation. Taken together, these results demonstrate that KAP1 deacetylation, which is regulated by SIRT1, is independent from ATM-induced KAP1 phosphorylation and has no effect on KAP1’s transcriptional co-repressor activity.

### Deacetylation promotes KAP1 function in NHEJ-mediated repair pathway

**Fig 4:**
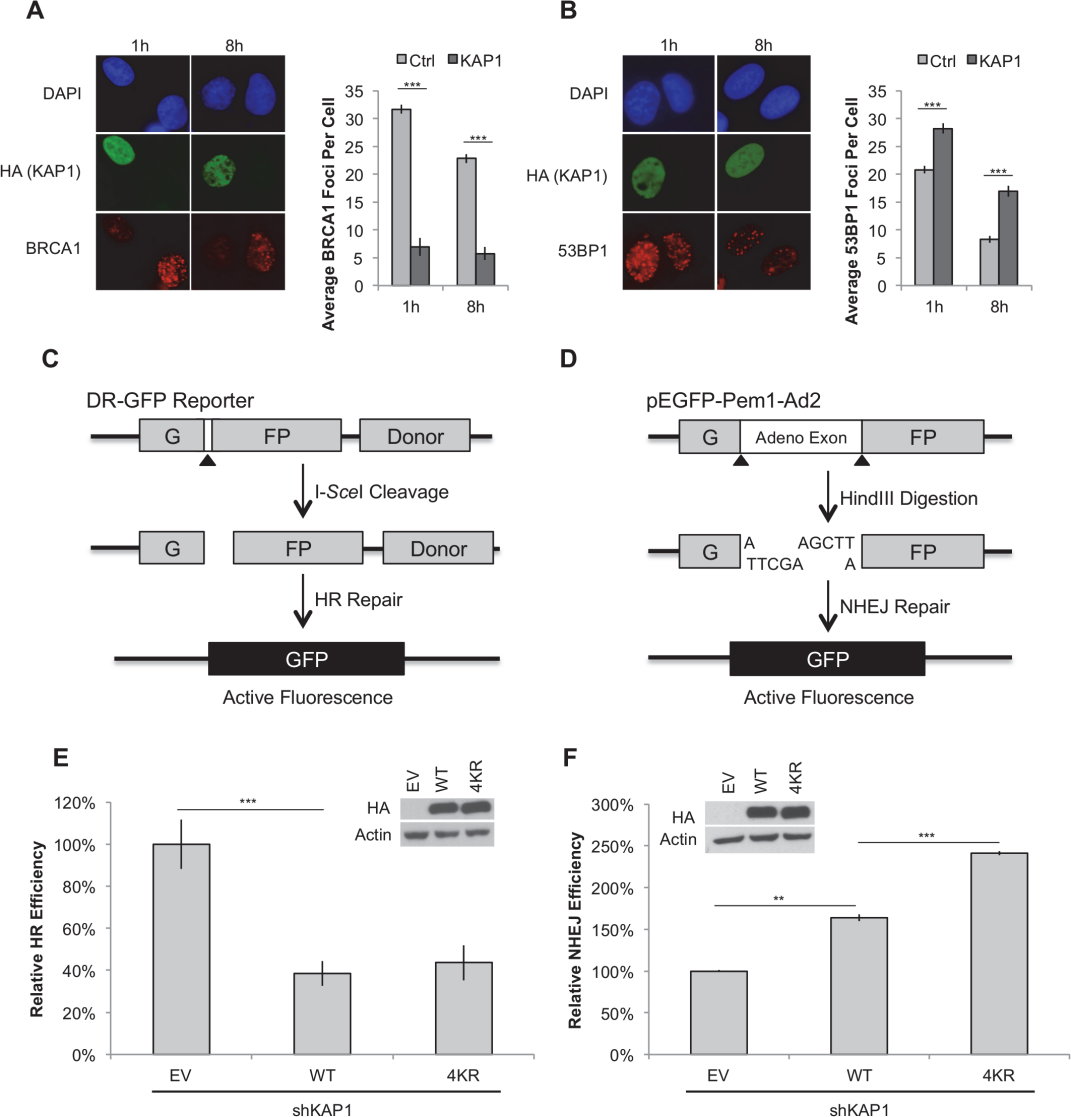
Deacetylation promotes KAP1 function in NHEJ-mediated repair. (A and B) HA-tagged KAP1 transfected U2OS cells were fixed at 1 hour and 8 hours following 2Gy-IR. BRCA1 and 53BP1 focus formation was assessed by immunostaining. The right panel shows the quantification of average foci per cell. (C) The DR-GFP reporter consists of a direct repeat of a mutated *GFP* gene, which is separated by one I-*Sce*I cleavage site, and a wild-type donor sequence as the template for HR repair. Successful repair of I-*Sce*I induces DNA cleavage by HR-mediated pathway results in GFP-positive cells, and therefore the HR efficiency can be quantified by flow cytometric analysis. (D) pEGFP-Pem1-Ad2 reporter contains a direct repeat of *GFP* gene, which is interrupted by an insertion of adenovirus exon. Pre-digestion of HindIII removes the inserted exon and create a DNA breakage on the reporter. NHEJ-mediated pathway re-ligates the DNA lesion and results in GFP-positive cells. NHEJ repair efficiency can then be quantified by flow cytometric analysis. (E) KAP1 was depleted by 3’UTR targeting shRNA in HEK293T cells. DR-GFP and I-*Sce*I were co-transfected with HA-tagged WT-KAP1 or 4KR mutant, and the HR repair efficiency was quantified at 48 hours post transfection. (F) Linearized NHEJ reporter was co-transfected with WT-KAP1 or 4KR mutant into KAP1 depleted cells, and the NHEJ repair efficiency was quantified at 48 hours post transfection. ** P≤0.01, *** P≤0.001.

SIRT1 facilitates DNA damage repair and promotes cell survival by deacetylating repair factors such as Ku70, NBS1, and XPA [52,53,55,72]. In addition to increasing the DNA repair capacity, SIRT1 also plays a distinct role in DSB repair pathway selection [56,58,59]. Interestingly, KAP1 has been reported to be involved in HR and NHEJ-mediated repair [80,81]. Based on these findings, we were interested in whether SIRT1-dependent KAP1 deacetylation plays a role in DSB repair pathways. First, we ectopically expressed HA-tagged KAP1 in U2OS cells and examined the recruitment of BRCA1 and 53BP1 to the DSB sites via immunostaining. To our surprise, we observed that up-regulation of KAP1 in these cells compromised the recruitment of BRCA1 to the IR-induced DSB sites, which suggested that KAP1 might suppress HR-mediated repair pathway (Fig 4A). On the contrary, higher KAP1 levels enhanced the formation of 53BP1 foci in response to DNA damage, suggesting that KAP1 might facilitate NHEJ-mediated repair process (Fig 4B).

To explore whether SIRT1-dependent KAP1 deacetylation plays a role in regulating KAP1’s function in different repair pathways, we depleted KAP1 by shRNA (S1 Fig) and followed by quantifying HR and NHEJ efficiency using the well-established reporter assay (Fig 4C and 4D) [69,70]. Consistent with our observations in immunofluorescence, reconstitution of WT-KAP1 significantly reduced HR-mediated repair capability (Fig 4E). However, we found that both WT-KAP1 and 4KR mutant showed similar inhibitory effect on HR, suggesting that the effect of KAP1 deacetylation in HR may be mild. Interestingly, when we examined the role of KAP1 in NHEJ-mediated repair pathway, we found that while cells expressing WT-KAP1 increased the efficiency of NHEJ-mediated repair, the expression of the 4KR mutant further promoted NHEJ (Fig 4F). Collectively, these results demonstrate that while KAP1 is a negative regulator of HR-mediated repair pathway, it can increase NHEJ efficiency. Furthermore, the positive effect of KAP1 in NHEJ repair can be further enhanced by SIRT1-dependent deacetylation.

### SIRT1-dependent KAP1 deacetylation enhances 53BP1 focus formation on DSB sites

**Fig 5:**
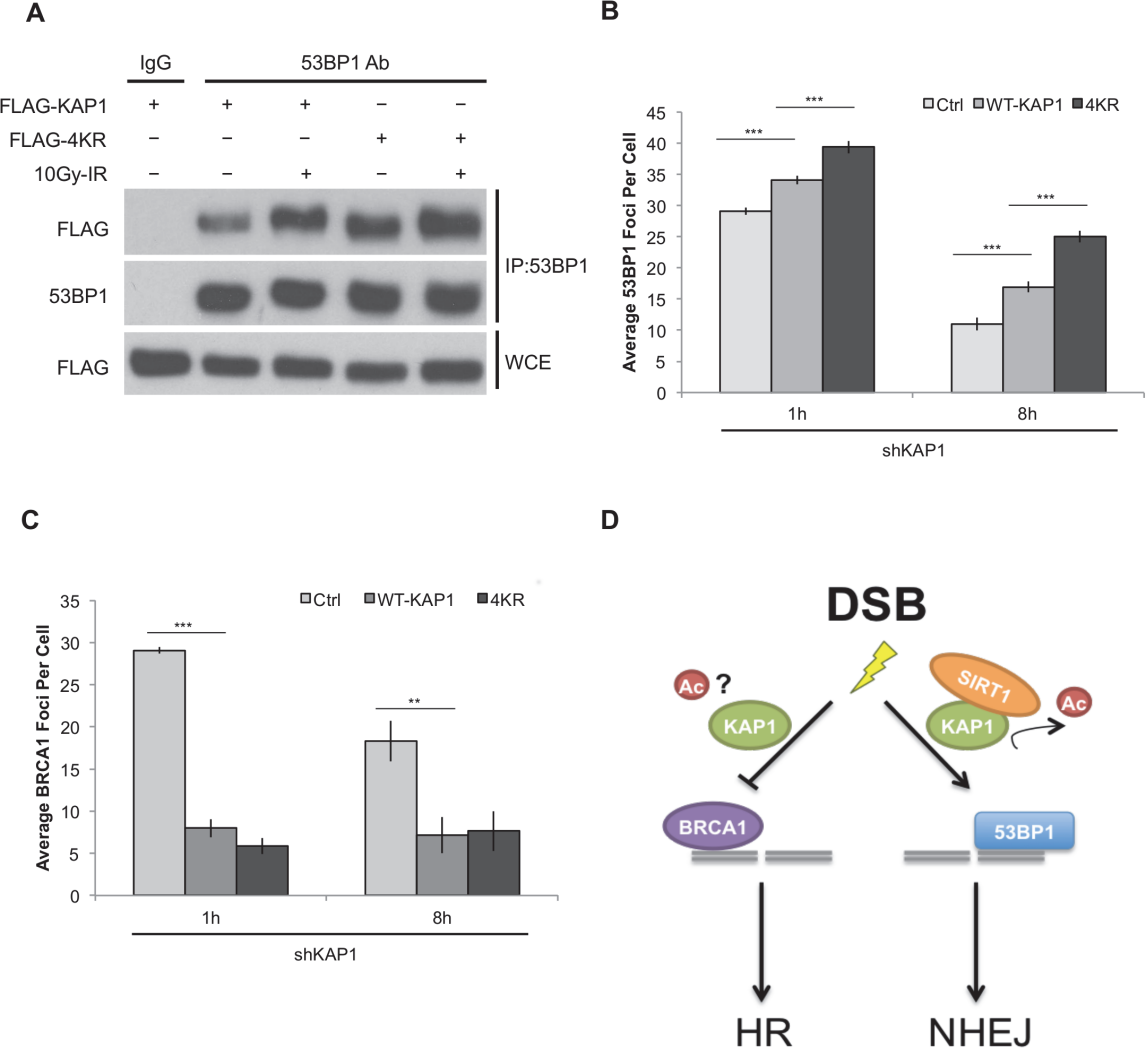
4KR mutant stabilizes the formation of 53BP1 foci on DSB sites. (A) FLAG-tagged WT-KAP1 or 4KR mutant were transfected into KAP1 depleted HEK293T cells. Cells were harvested 1 hour following 10Gy-IR, and cell lysates were used for anti-53BP1 immunoprecipitation. Protein-protein interaction between 53BP1 and KAP1 was determined by immunoblotting. (B and C) Quantifications of 53BP1 and BRCA1 focus formation in HA-tagged WT-KAP1 or 4KR reconstituted U2OS cells (assessed 1 hour and 8 hours after 2Gy-IR). (D) Model of SIRT1-KAP1 regulatory mechanism in DSB repair pathways. ** P≤0.01, *** P≤0.001.

We next explored the mechanism by which KAP1 deacetylation regulates NHEJ. As a critical NHEJ promoting factor, 53BP1 is essential for KAP1 recruitment to the DNA damage sites [82]. Consistent with the previous findings, we observed enhanced protein-protein interaction between 53BP1 and KAP1 after IR stimulation (Fig 5A). Interestingly, compared to the WT-KAP1, we found stronger binding affinity between 53BP1 and 4KR mutant, indicating that KAP1 deacetylation might function in DNA damage repair through 53BP1 related pathways. In addition, when we examined the recruitment of 53BP1 to the breakage site, we found that cells reconstituted with 4KR mutant showed increased formation of 53BP1 foci compared to WT-KAP1 reconstitution (Fig 5B), suggesting that KAP1 deacetylation by SIRT1 might be a mechanism to promote NHEJ-mediated repair pathway through 53BP1. We also found that KAP1 reconstitution suppressed the recruitment of BRCA1; however, no significant difference was found between WT-KAP1 and 4KR expressed cells (Fig 5C). In summary, these results demonstrate that KAP1 plays an important role in balancing DNA repair pathways by inhibiting HR and promoting NHEJ. Moreover, SIRT1 contributes to NHEJ pathway by deacetylating KAP1, and therefore stabilizes the association between KAP1 and 53BP1, which leads to increased formation of 53BP1 foci in response to DNA damage (Fig 5D).

## Discussion

As a master regulator of the genome, KAP1 has been implicated in epigenetic modifications and chromatin condensation. It is known that the post-translational modifications, such as phosphorylation and SUMOylation, play key roles in regulating KAP1 function to execute pleiotropic effects. To our knowledge, the findings presented here are the first to characterize the functional significance of KAP1 acetylation. We demonstrated KAP1 as a regulator for DSB repair pathway choice by inhibiting HR while promoting NHEJ-mediated repair. Importantly, we unambiguously established KAP1 as a target of SIRT1 and that the deacetylation of KAP1 on Lys266, Lys377, Lys469, and Lys770 promotes NHEJ, presumably by stabilizing the formation of 53BP1 foci in response to DSB.

Previous studies have suggested the functional significance of acetylation in coordinating the dynamics of the DDR. For example, H3K56 deacetylation by HDAC1/2 promotes the persistence of NHEJ repairing factors at the DSB sites [83]. On the other hand, MOF-mediated H4K16 acetylation is important for MDC1, 53BP1, and BRCA1 recruitment to DNA damage sites [84]. In addition to histone modification, SIRT6-mediated deacetylation of CtIP, a DNA endonuclease, stimulates recruitment of Rad51 and replication protein A (RPA) to the breakage sites, which results in activation of ATR signaling and HR-mediated repair pathway [85]. Furthermore, acetylation of ATM Lys-3016 by Tip60 activates ATM activity in response to DNA damage [86]. These reports have demonstrated that acetylation can influence the DDR factor assembly and promote DNA repair. Our study has established an acetylation-mediated signaling for 53BP1 recruitment through a SIRT1-KAP1 regulatory mechanism, which extends and complements the understanding of acetylation in DNA damage repair.

When cells are stressed by DNA damage sources, the insult generates DSBs that activates ATM kinase to promptly phosphorylate Ser824 on KAP1 in the vicinity of the damage sites [87]. Given that KAP1 is a major chromatin modifier, one plausible function of KAP1 in DNA repair is through modulation of local chromatin architecture. Indeed, the robust KAP1 pSer824 causes a transient chromatin relaxation, which renders the chromatin to be more susceptible to nuclease digestion *in vitro* [64]. KAP1 pSer824 has also been proposed to diminish the auto-SUMOylation in its PHD domain and lead to downstream target gene expression [65,66,77], which may indicate the de-condensation of chromatin structure. In addition, previous reports also showed that phosphorylation and SUMOylation regulate KAP1’s function in NHEJ and HR [80,81]. However, since no apparent change in chromatin superstructure or the intrinsic KAP1 mobility was identified [64,76,82], it is unclear whether KAP1 regulates the DDR solely through the alteration of chromatin superstructure. It is noteworthy that we didn’t observe crosstalk between KAP1 pSer824 and acetylation. In addition, the 4KR acetylation mutant showed similar activity in transcriptional repression when compared to WT-KAP1. These findings demonstrate that KAP1 deacetylation may have minimal effects in regulating local chromatin environment. On the other hand, we propose that KAP1 deacetylation enhances its binding to 53BP1 and sustains the formation of 53BP1 foci on the DSB sites, which leads to increased NHEJ efficiency. Although reconstitution of WT or 4KR mutant did not show apparent difference on HR, previous studies have suggested that KAP1 plays an essential role in HR-mediated DSB repair [81,88]. Since HR-mediated repair pathway is only predominant in G2 and S phase, it is possible that the effect of 4KR mutant in HR repair was less significant in the unsynchronized condition. Therefore, we hesitate to conclude that this SIRT1-KAP1 regulatory mechanism is specific to NHEJ repair. Collectively, these studies suggest that in addition to chromatin modulation, KAP1 may also promote the DDR through a novel regulatory mechanism by influencing the DDR complex assembly. Future work will be required to address whether the SIRT1-KAP1 regulatory mechanism affects DSB repair in different cell cycle phases. In addition, it will be interesting to investigate if there is a collaborative effect between KAP1-dependent chromatin regulation and the DDR factor assembly, which will further elucidate the role of KAP1 in maintaining genome integrity.

SIRT1 regulates DNA damage repair at different levels. First, SIRT1 deficiency impairs the focus formation of γ-H2AX, BRCA1, Rad51, and NBS1 in response to IR [89], suggesting its role in the DDR signaling activation and processing. Second, enhanced interaction between SIRT1 and WRN helicase after DNA damage promotes the long-range DNA end resection, which facilitates HR efficiency [57,90–92]. Third, deacetylation of Ku70 by SIRT1 prevents the translocation of pro-apoptotic factor BAX to mitochondria, thereby blocking mitochondrial apoptosis and inducing Ku70-dependent NHEJ repair pathway [52,93]. These studies have demonstrated the significance of SIRT1 in the DDR factor assembly and the DDR signaling activation. However, it is worth noting that SIRT1 recruitment causes reduction of euchromatic markers H4K16Ac, H3K9Ac, and H1K26Ac [94], which results in chromatin compaction. Moreover, SIRT1 deacetylates Suv39h1 to elevate its enzyme activity, and therefore promotes the spreading of heterochromatic makers H3K9me3 [46]. Since heterochromatic regions are refractory to the activation of the DDR signaling, the role of SIRT1 in the DDR is contradictory to its function in chromatin condensation. Our findings show that SIRT1 can deacetylate heterochromatic factor KAP1 and increase the efficiency of 53BP1 recruitment to the damage site. This SIRT1-KAP1 regulatory mechanism may compensate for decreased repair efficiency caused by SIRT1-dependent infrastructural barrier. Hence, by identifying KAP1 as a novel downstream effector of SIRT1, our study will help reveal a mechanism that fine-tune the biological function of SIRT1 in the DDR and heterochromatic repair.

In conclusion, our results establish the functional significance of KAP1 deacetylation in the DDR. We propose that SIRT1 deacetylates KAP1 (Lys266, Lys377, Lys469, and Lys770) and enhances the interaction of KAP1-53BP1 after DNA damage. This stabilized protein-protein interaction sustains the 53BP1 focus formation and promotes NHEJ-mediated DNA repair. Given that the SIRT1-regulated deacetylation does not crosstalk with pSer824 on KAP1, which is the only known for KAP1-dependent chromatin de-condensation to date, our work highlights a potential SIRT1-KAP1 regulatory mechanism for DSB repair that is independent from modulating the infrastructure of the chromatin.

## Supporting Information

S1 Fig KAP1 3UTR’ targeting shRNA. (PDF)

S1 Table Primers for 4KR mutagenesis. (PDF)

S2 Table KAP1 alignment analysis. (PDF)
