# Supplementary figures and images for: KAP1 Deacetylation by SIRT1 Promotes Non-Homologous End-Joining Repair
Source: PLoS One. 2015 Apr 23;10(4):e0123935. doi: 10.1371/journal.pone.0123935 (PMC4408008; doi:10.1371/journal.pone.0123935)

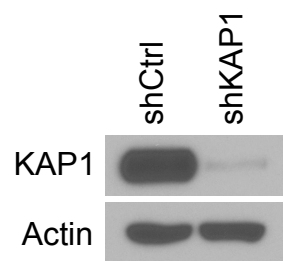

Supplement: S1 Fig — (PDF) [file pone.0123935.s001.pdf]
