# Supplementary material for: KAP1 Deacetylation by SIRT1 Promotes Non-Homologous End-Joining Repair
Source: PLoS One. 2015 Apr 23;10(4):e0123935. doi: 10.1371/journal.pone.0123935 (PMC4408008; doi:10.1371/journal.pone.0123935)
Supplement: S1 Table — (PDF) [file pone.0123935.s002.pdf]

K266R	Forward	5’-GAAGCGCCTTGGGGACAGACATGCAACATTGCAGA-3’
	Reverse	5’-TCTGCAATGTTGCATGTCTGTCCCCAAGGCGCTTC-3’
K377R	Forward	5’-GCTGCACCGGGCCCTCAGGATGATTGTGGATCCCG-3’
	Reverse	5’-CGGGATCCACAATCATCCTGAGGGCCCGGTGCAGC-3’
K469R	Forward	5’-CATGTGTCAGGTGTGAGACGGTCCCGCTCAGGT-3’
	Reverse	5’-ACCTGAGCGGGACCGTCTCACACCTGACACATG-3’
K770R	Forward	5’-GGATGTGGGCCGCATGTTCAGGCAATTCAACAAGTTAACTG-3’
	Reverse	5’-CAGTTAACTTGTTGAATTGCCTGAACATGCGGCCCACATCC-3’
