# Supplementary material for: KAP1 Deacetylation by SIRT1 Promotes Non-Homologous End-Joining Repair
Source: PLoS One. 2015 Apr 23;10(4):e0123935. doi: 10.1371/journal.pone.0123935 (PMC4408008; doi:10.1371/journal.pone.0123935)
Supplement: S2 Table — (PDF) [file pone.0123935.s003.pdf]

```
                                       K266
Human           EDAVR-NQRKLLASLVKRLGDKHATLQKSTKEVRSSIRQVSDVQKRVQVDVKMAILQIMK 304
Mouse           EDAVR-NQRKLLASLVKRLGDKHATLQKNTKEVRSSIRQVSDVQKRVQVDVKMAILQIMK 305
Sheep           EDAVR-NQRKLLASLVKRLGDKHATLQKNTKEVRSSIRQVSDVQKRVQVDVKMAILQIMK 305
Cattle          EDAVR-NQRKLLASLVKRLGDKHATLQKNTKEVRSSIRQVSDVQKRVQVDVKMAILQIMK 284
Cat             EDAVR-NQRKLLASLVKRLGDKHATLQKNTKEVRSSIRQVSDVQKRVQVDVKMAILQIMK 171
Frog            EDAVK-NQRKVLSALVKRLGDKHTALQKSTKDVRTSIRQVSDLQKRLQVDVKMAILHIMK 253
Zebrafish       DEACQ-NQKGIIATFMTKLQEKRGLVEYSASEVQKRLKEVAETHKKVEHEIKIAVFTLIN 375

                            K377
Human           KKLIYFQLHRALKMIVDPVEPH-----GEMKFQWDLNAWTKSAEAFGKIVAERPGTNSTG 419
Mouse           KKLIYFQLHRALKMIVDPVEPH-----GEMKFQWDLNAWTKSAEAFGKIVAERPGTNSTG 420
Sheep           KKLIYFQLHRALKMIVDPVEPH-----GEMKFQWDLNAWTKSAEAFGKIVAERPGTNSTG 420
Cattle          KKLIYFQLHRALKMIVDPVEPH-----GEMKFQWDLNAWTKSAEAFGKIVAERPGTNSTG 399
Cat             KKLIYFQLHRALKMIVDPVEPH-----GEMKFQWDLNAWTKSAEAFGKIVAERPGTNSTG 286
Frog            KKLIHFQLHRALKVIVDPVEPL-----GDLKFQWDSDTWTKHAEFFGKIVCDKTGVPQSS 368
Zebrafish       KRLITYQLRLIMKARVDAVPPAN----GAVRFFCDPTFWAKNVVNLGNLVIEKVAPTAPP 491

                                                          K469
Human           --------------MEVQEGYGFGSGDDPYSSAEPHVSGVKRSRSGEGEVSGLMRKVPRV 488
Mouse           --------------MEVQEGYGFGS-DDPYSSAEPHVSGMKRSRSGEGEVSGLLRKVPRV 488
Sheep           --------------MDVQEGYGFGS-DDPYSSAEPHVSGVKRPRSGDGEVSGLMRKVPRV 488
Cattle          --------------MEVQEGYGFGS-DDPYSSAEPHVSGVKRPRSGDGEVSGLMRKVPRV 467
Cat             --------------MEVQEGYGFGS-DDPYSSAEPHVSGVKRSRSGEGEVSGLMRKVPRV 354
Frog            --------------MQVQV-YGFCAFEGESSGFDTFP-GQKRGRSSEGDVNELLKKVPRV 428
Zebrafish       AAIAQKHQQQHQHHQQQQHQHQHQQQQQQQQQQQQQQQQQQQQQHQQQIQQQMRIASQM 586

                                 K770
Human           PYSSPQEFAQDVGRMFKQFNKLTEDKADVQSIIGLQR----------------------- 790
Mouse           PYSSPQEFAQDVGRMFKQFNKLTEDKADVQSIIGLQR----------------------- 790
Sheep           PYSSPQEFAQDVGRMFKQFNKLTEDKADVQSIIGLQR----------------------- 790
Cattle          PYSSPQEFAQDVGRMFKQFNKLTEDKADVQSIIGLQR----------------------- 769
Cat             PYSSPQEFAQDVGRMFKQFNKLTEDKADVQSIIGLQR----------------------- 656
Frog            SYSTPEDFVRDVWMMFRSVSKLAEDKAVVQSIIELQS----------------------- 716
Zebrafish       HYKSPKEFVSDVRLVFSNCAKYNEMSRIIQVYDEEKQSNVQADSEVAE-----------A 1099
```
